# Digital mapping and spatial characteristics analyses of heavy metal content in reclaimed soil of industrial and mining abandoned land

**DOI:** 10.1038/s41598-018-35624-9

**Published:** 2018-11-21

**Authors:** Shiwen Zhang, Huilin Liu, Ming Luo, Xu Zhou, Mei Lei, Yuanfang Huang, Yan Zhou, Chang Ge

**Affiliations:** 1 College of Earth and Environmental Sciences, Anhui University of Science and Technology, Huainan, 232001 China; 2 Land Management Center of Ministry of Land and Resources of the People’s Republic of China, Beijing, 100035 China; 3 Institute of Geographical Sciences and Natural Resources Research, Beijing, 100101 China; 4 College of Resources and Environmental Science, China Agricultural University, Beijing, 100193 China

## Abstract

The reclaimed soil properties of industrial and mining wasteland have strong spatial specificity. The paper aimed to screen out a hybrid multifractal and kriging (Named as Mkriging) method for digital mapping and scientifically reveal the spatial distribution characteristics in view of heavy metal in reclaimed soil of industrial and mining abandoned land. The results of the study showed that for reasons of history and reclamation, the original samples of heavy metals in reclaimed soil of industrial and mining abandoned land showed a very large range and variation degree, the *C*_0_/(*C*_0_ + *C*_1_) values of different heavy metals basically were all greater than 50%, random factors played a dominant role. The five kinds of heavy metals in reclaimed soil were in the following descending order in terms of homogeneity: Cd, As, Hg, Ni and Cr. Compared with ordinary Kriging method, the relative improvement of root mean squared errors of elements Cd, Hg, As, Cr and Ni based on Mkriging were 95.28%, 61.74%, 78.54%, 82.51% and 83.58% respectively. The higher the fractal degree of heavy metals in reclaimed soil was, the higher the prediction accuracy will be. Mkriging method is more suitable for spatial prediction of heavy metals in reclaimed soil of industrial and mining abandoned land.

## Introduction

Industrial and mining abandoned land is a special type of land space damaged due to industrial and mining activities. Its reclamation and utilization play an important role in ameliorating ecological environment, optimizing the development and layout of land space and promoting resource conservation and ecological civilization. The digital mapping and spatial characteristics analyses of soil properties in the reclaimed area are an important link of reclamation monitoring.

Due to the long indigenous smelting sulfur, the industrial and mining abandoned land in the southwestern region of China were seriously polluted by heavy metals, which is one of the obstacles holding back the progress of ecological civilization, leads to the reduction of yield and quality of the crops on the reclaimed land and threatens the safety of the ecosystem and humans in the reclaimed area^1^. A lot of research has been done on heavy metals in soil at home and abroad^2–24^. Currently, the research focuses on the non-restructured soil in cities, vegetable farms, orchards, and farmland near a mining area^6,7,11,20^, and analyzes and evaluates the pollution condition in soil and their impact on safe land use based on soil environment standard or regional geological background from the perspective of sampling points^2–24^. There is a complicated historical background in the wasteland of industrial and mining. There are significant differences in the reclamation project. The heavy metal content in the rebuilt soil is affected by the terrain and human factors. Even in a very small distance, there may be a great change. The variability and variability are strong. A conventional and regular Kriging method requires minimum error variance and has a very strong smoothing effect, which will reduce or eliminate the areas with strong spatial variability and makes the whole study area tend to a median (or interval). It is impossible to realize the conversion of heavy metals from the point to surface in the reclamation soil, to influence the follow-up monitoring, evaluation and selection of management and protection measures. Therefore, this article aims to find a digital mapping method that can not only highlight specific values and effectively maintain and reproduce the spatial structure of original data-based variables but also guarantee the overall accuracy and model fitting effect of the prediction result. Mkriging method is in fact a kind of extended interpolation of moving weight average, which is multiplied by a singularity index as correction factor since interpolation of original moving weight average. It overcomes the impact of smoothing effect, and properly maintains the spatial variation degree. At present, the multifractal theory has been applied in mineral research, agricultural research and other nonlinear spatial analysis^25–31^, but its application in digital mapping and spatial distribution characteristics analyses of heavy metals in soil, particularly in reclaimed soil of industrial and mining abandoned land, is seldom reported.

This article chose heavy metals in reclaimed soil of industrial and mining abandoned land in the southwestern region of China as a study object and carried out the following research: (1) Using Mkriging method, the spatial mapping of heavy metals in reclaimed soils from industrial and mining wastelands was realized on the basis of variogram analysis and multifractal analysis; (2) Reveal the spatial distribution characteristics of heavy metal content in reclaimed soil of industrial and mining abandoned land. The above research will provide methodological guidance for spatial prediction of heavy metal content in reclaimed soil from industrial wasteland.

## Overview of the Study Area and Data Processing

### Overview of the study area

The study area lies in the southwestern region of China, and began mining and beneficiation of sulfur ore in the late 1950s. In 2004, the mine was bankrupt, restructured and handed over to local township government for management. After 40 years’ mining and smelting, solid waste (such as sulfur slag) accumulation is very large. The digging, occupation and pollution of the mining area are serious, and the ecological environment of the whole mining area is harsh. The total reclaimed area is 266.49 hm^2^. The work of reclamation was initiated in 2013 and completed with acceptance in 2014. The study area, with the altitude being 500~1100 m, is in the transition belt from the southern margin of Sichuan Basin to Guizhou Plateau, and within Chishui River basin. The study area bears the climatic features of Sichuan Basin and Guizhou Plateau. The type of soil is yellow soil.

### Data acquisition and processing

**Figure 1:**
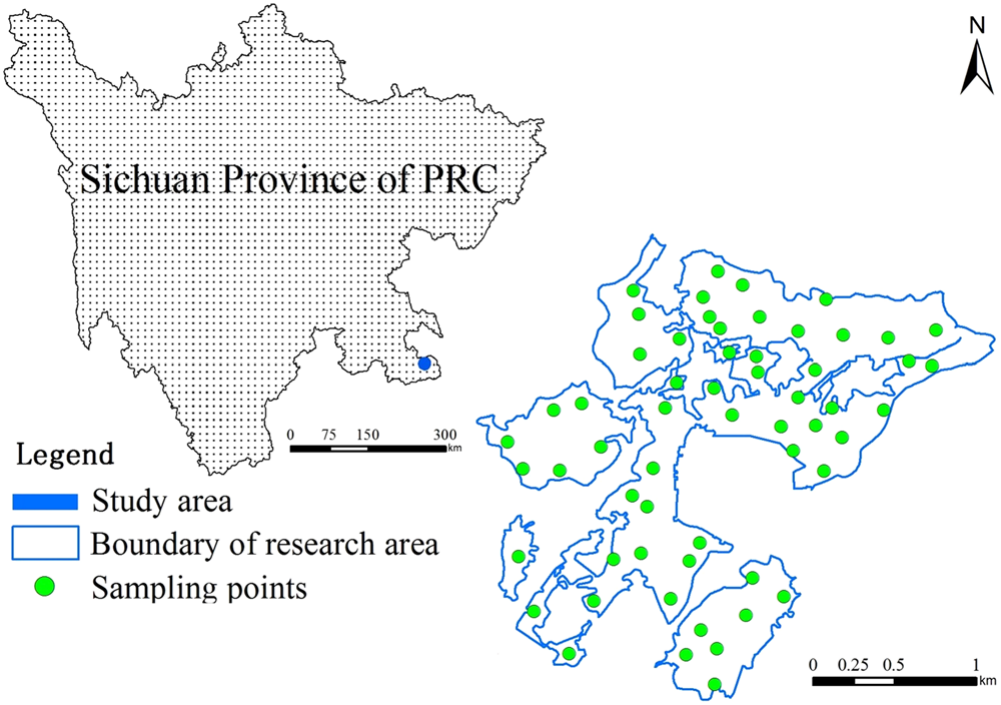
Map of location and sampling points of the study area. The graph was made by ArcGIS10.2, specific operation process can be further reference to http://www.esrichina.com.cn/sofewareproduct/ArcGIS/.

**Figure 2:**
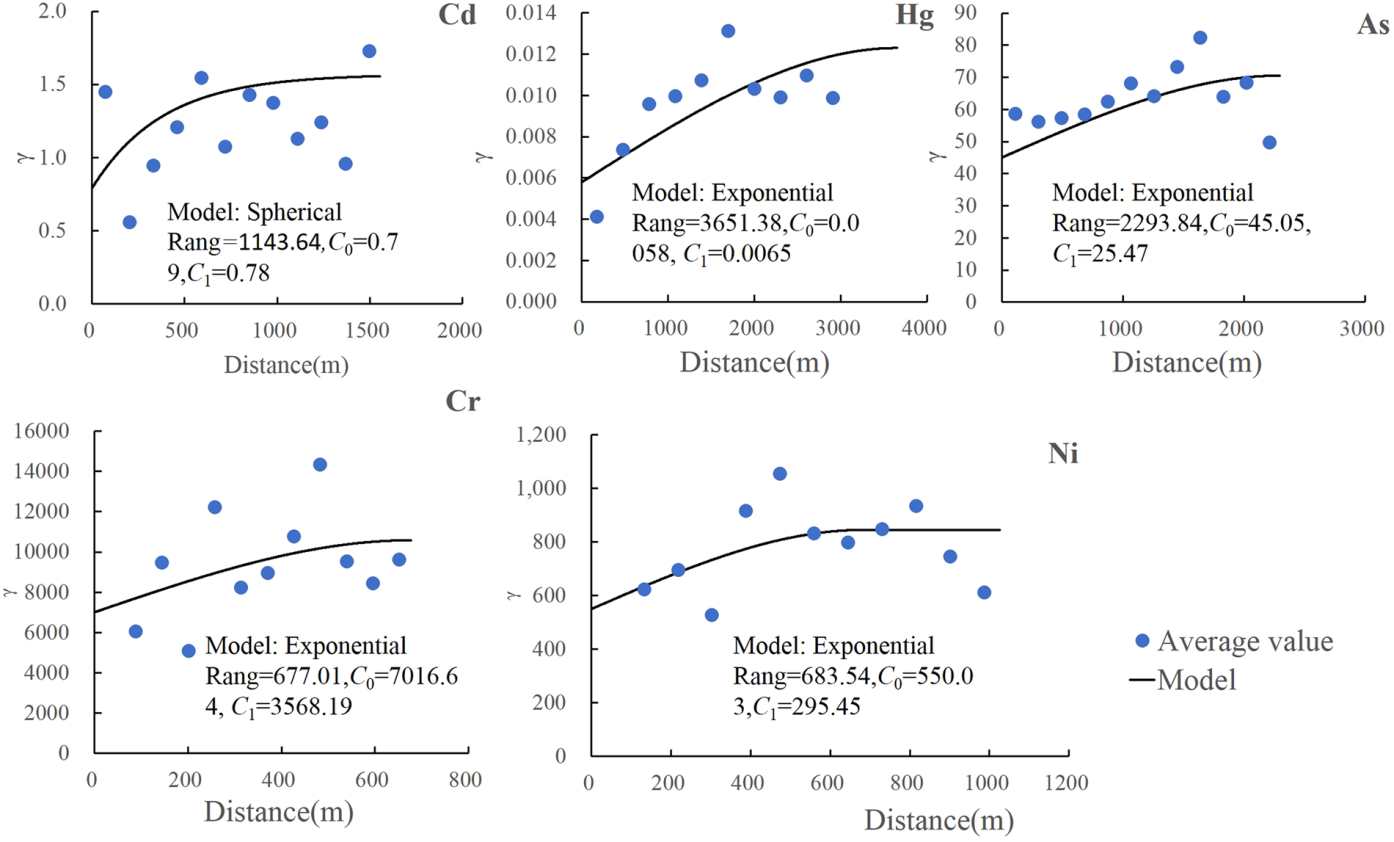
Variogram functions and their characteristic values of different heavy metal. Note: The dashed in Fig. 2 were model results that provide the best fit for the data points. Binned values are shown as circles, triangles, meters, and crosses, and were generated by grouping (binning) empirical semivariogram points together using square cells that are one lag wide. Nugget(*C*_0_) represents spatial variability due to random errors such as measurement errors and microscale processes; Partial sill(*C*_1_) is the structural variance, which indicates the spatial structure caused by spatial correlation.

Soil samples were taken in July 2016. A total of 58 samples were selected to represent a range of reclamation measures and other factors (Fig. 1). The interval between all the samples is less than 500 m, which is within the range of the semi-variogram of each heavy metal (Fig. 2). The sampling depth was 0~20 cm. The locations of the sampling sites were recorded using a global positioning system (GPS) receiver (Garmin3790T). Three to five soil samples were collected at depth 0–25 cm over a circle of radius 10 m surrounding the specified sampling location and mixed thoroughly. About 1.5 kg soil was collected from each mixed sample. According to the relevant standards such as Soil Quality-Determination of Lead, Cadmium-Graphite Furnace Atomic Absorption Spectrophotometry (GB/T17141-1997), Soil and Sediment-Determination of Mercury, Arsenic, Selenium, Bismuth, Antimony-Microwave Dissolution/Atomic Fluorescence Spectrometry (HJ 680-2013), and Soil and Sediment-Determination of Mercury, Arsenic, Selenium, Bismuth, Antimony-Microwave Dissolution/Atomic Fluorescence Spectrometry (HJ 680-2013) and so on, five types of heavy metals in the reclaimed soil, including cadmium (Cd, mg/kg), arsenic (As, mg/kg), chromium (Cr, mg/kg), mercury (Hg, mg/kg) and nickel (Ni, mg/kg) were tested.

## Methods

### Methods of digital mapping and spatial distribution characteristics analyses

#### Geostatistics

Geostatistics is a science based on the theory of regionalized variable and using variogram function as a main tool to study natural phenomena. The ordinary Kriging method uses spatial autocorrelation to estimate the value of the estimated point in the minimum variance.

1 $$z({x}_{0},{y}_{0})=\sum _{i=1}^{n}{\lambda }_{i}\cdot {z}_{i}$$

2 $$\{\begin{array}{c}\sum _{i=1}^{n}{\lambda }_{i}\gamma ({x}_{i}-{x}_{j})+\mu =\gamma ({x}_{j}-{x}_{0}),\,\,j=1,2,3,\ldots ,n\\ \sum _{i=1}^{n}{\lambda }_{i}=1\end{array}$$

where, *z*(*x*_0_, *y*_0_) is the value of the estimated point, *n* is the number of adjacent related points, and λ_*i*_ is the kriging weights. *γ*(*x*_*i*_ − *x*_*j*_) is the value of variation function between *x*_*i*_ and *x*_*j*_ and *μ* is the Lagrange multiplier. By solving the above linear equations, all the weights *λ*_*i*_ and Lagrange multipliers *μ* can be obtained. This article chose ordinary Kriging as a control method. For more information, please refer to related literature^32–47^.

#### Multifractal analysis

Multifractal analysis method firstly obtains the probability distribution of unevenly distributed spatial variables (Heavy metal of reclaimed soil in the article) by box-counting method through a computer.

As for any given real number *q*, a partition function *χ*_*q*_(*l*) may be defined, and probability *μ*_*i*_(*l*) is weighted and summated through the *q*^th^ power.

Its mathematical expression is:

3 $${\chi }_{q}(l)=\sum _{i=1}^{N(l)}{\mu }_{i}^{q}(l)\,(\,-\,\infty  < q < \infty )$$

where *l* is measurement scale (side length of the box); *N*(*l*) is the number of boxes; *μ*_*i*_(*l*) is probability distribution of spatial variable.

If spatial variable bears a fractal feature, then *χ*_*q*_(*l*) ≈ *l*^*τ*(*q*)^, i.e., partition function *χ*_*q*_(*l*) and *l* have a relation of power function. *τ*(*q*) generally refers to a quality index. The singular exponent *α* and multifractal spectrum *f*(*α*) can be further calculated.

4 $$\alpha (q)=\frac{{\rm{d}}\tau (q)}{{\rm{d}}q}$$

5 $$f(\alpha (q))=\alpha (q)q-\tau (q)$$

The *τ*(*q*) and *q* are transformed into *f* (*α*) and *α* through the Eqs 4 and 5. Multifractal spectrum width Δ*α* and Rayleigh dimension *D*_*q*_ can be further calculated through the following formula^48–51^.

6 $${\rm{\Delta }}\alpha ={\alpha }_{\max }-{\alpha }_{\min }$$

7 $${D}_{q}=\mathop{\mathrm{lim}}\limits_{\varepsilon \to 0}\frac{\mathrm{ln}\,{\chi }_{q}(l)}{(q-1)\mathrm{ln}\,l}$$

where *α*_max_ and *α*_min_ is the maximum and minimum value of the singular exponent, respectively.

#### Mkriging

Considering the spatial distribution characteristics of the properties of reclaimed soil, we combined fractal theory with ordinary Kriging (OK) and conducted spatial prediction of quality elements of reclaimed land by Multifractal Kriging (Named as Mkriging) method to eliminate the impact of the smoothing effect on prediction result. Mkriging is an extended moving weighted average interpolation method. It uses the result of moving weighted average times a factor relevant with measurement scale and singularity exponent as an estimated value of the regionalized variable. Usually, the mean aggregation of spatial variable varies with measurement scale^28,52–56^.

The relation between the measure and the dimension of a small square with a length of *l* is like a formula (8).

8 $$f({x}_{0},l)\cdot l=b({x}_{0})\cdot {l}^{\alpha ({x}_{0})}$$

where, *α*(*x*_0_) is the singularity exponent at point *x*_0_. b is a constant.

Taking the square with a length of *l* as the center, the measure in the square of *Nl* is:

9 $$Nl\cdot \overline{{Z}_{N}}=b\cdot {(Nl)}^{\alpha }$$

Simultaneous Equations (8) and (9) can be obtained:

10 $${Z}_{i}={N}^{2-\alpha }\cdot \overline{{Z}_{Nl}}$$

If the mean value in the square of *Nl* is estimated by formula (1), the multidimensional fractal Kriging estimation formula in two-dimensional case can be obtained.

11 $$Z({x}_{0},{y}_{0})={N}^{2-\alpha }\cdot \sum _{i=1}^{n}{\lambda }_{i}\cdot {Z}_{i}$$

Geostatistical analysis, multifractal analysis and spatial digital mapping were completed in the MATLAB and ArcGIS10.2 software.

### Analysis on the effect of spatial digital mapping

We evaluated the mapping effect from three aspects: reproducibility, prediction accuracy and model simulation effect. We used the coverage diagram and coverage ratio of specific value (CRSV) to reflect the reproducibility. We formed a maximum (minimum) specific value group consisting of 20% of the maximum (minimum) values of measured values and predicted values. The CRSV value is the coincidence percentage of coverage of minimum (maximum) specific value of measured values and predicted values. The higher the percentage is, the better the effect of spatial prediction will be^54^. A cross validation method was adopted to validate the simulation accuracy and model fitting effect. Root mean squared errors (RMSE) and mean squared deviation ratio (MSDR) were adopted to validate the spatial prediction accuracy and model simulation effect. They are defined as:

12 $${\rm{RMSE}}=\sqrt{\frac{1}{n}{\sum _{i=1}^{n}\{(z({x}_{i})-\mathop{z}\limits^{\frown {}}({x}_{i}))\}}^{2}}$$

13 $${\rm{MSDR}}=\frac{1}{n}\sum _{i=1}^{n}(\frac{{\{(z({x}_{i})-\mathop{z}\limits^{\frown {}}({x}_{i}))\}}^{2}}{{\sigma }_{i}^{2}})$$

where *z*(*x*_*i*_) is the measured value, $\mathop{z}\limits^{\frown {}}({x}_{i})$ is the predicted values, *σ*_*i*_ and *n* are the variance and the number of samples in the validation set, respectively. RMSE was used to evaluate the prediction accuracy. The smaller the RMSE value is, the more accurate the prediction result will be. MSDR was used to evaluate the degree of fitting of theoretical variation function. The closer the MSDR value is to 1, the more accurate the fit variation function will be^45,47^.

## Results and Analyses

### Variogram analysis

The typical statistical characteristic values, optimum variogram functions and their relevant parameters of five heavy metals Cd, Hg, As, Cr and Ni in real samples were obtained based on the SPSS20.0 and ArcGIS10.2.

The full-sample amplitudes of heavy metal elements Cd, Hg, As, Cr and Ni in reclaimed soil were 6.01 mg/kg, 0.56 mg/kg, 28.74 mg/kg, 461.27 mg/kg and 145.70 mg/kg. The coefficients of variation (*CV*) of the above five kinds of heavy metals were 97.34%, 51.92%, 46.66%, 50.54% and 37.91% in turn, and the *CV* value of Cd was above 90%. There is mutability and strong spatial variability of heavy metal content of reclaimed soil of industrial and mining abandoned land from the variation and *CV*. This was mainly due to the difference of types and degrees of damage caused by the mining activities, and the difference of the reclaim measures and standards adopted in the process of reclamation.

Referring to the Technical Specification for Soil Environmental Monitoring, the variability coefficient and relative deviation are used to determine whether the sample points meet the research requirements. The formula is as follows.

14 $$n={t}^{2}\cdot CV/{m}^{2}$$

where, *n* is the number of samples; *t* is the value of t at a certain degree of freedom at a given confidence level (generally 95% for soil monitoring); *CV* is the coefficient of variation (%) obtained from the collected data; *m* is the acceptable relative deviation (%); and soil monitoring is generally limited to 10–20%.

According to the analysis results, the average coefficient of variation of all monitoring indicators was 56.87%, and the acceptable relative deviation m was taken as the minimum value of 10%. Through calculation, we need to set up sampling points 49. Based on the above results, combined with the variogram range and the area of the study area, the 58 sampling points meet the requirements.

Table 1 indicated that different types of heavy metals have different statistical distributions. The distribution characteristics of heavy metals in reclaimed soil decided that it was not advisable to adopt ordinary Kriging with strong smoothing and central effects (refers the situation that the smoothing action of the model makes values of simulation converge towards a narrow range), which is unable to meticulously depict the mutation law of some zones.

**Table 1:** The statistics characteristics of heavy metal content for reclamation soil of historical mining wasteland.

Elements	Rang (mg/kg)	Mean (mg/kg)	*CV*/%	*p*-values
Cd	6.01	1.18	97.34	0.02
logCd	—	—	—	0.119
Hg	0.56	0.21	51.92	0.763
As	28.74	17.22	46.66	0.602
Cr	461.27	186.15	50.54	0.002
logCr	—	—	—	0.135
Ni	145.70	70.88	37.91	0.052

Note: *CV* is the coefficients of variation. There is a normal distribution in *p* > 0.05 through KS test.

Nugget/sill ratio (*C*_0_/(*C*_0_ + *C*_1_)) refers to the ratio of spatial heterogeneity caused by random parts to total variation of the system and can usually be used to measure spatial correlation of variables. The smaller the ratio is, the stronger the spatial correlation will be. If the ratio is smaller than 25%, it means the variable has strong spatial correlation; if the ratio is 25~75%, it means moderate spatial correlation; if the ratio is greater than 75%, it means weak spatial correlation^43,44^. The nugget/sill ratios of heavy metals Cd, Hg, As, Cr and Ni in reclaimed soil were 50.32%, 47.15%, 63.88%, 66.29% and 65.06%, respectively, close to 75%, showing moderate spatial autocorrelation (Fig. 2). *C*_0_, *C*_1_ and *C*_0_/(*C*_0_ + *C*_1_) indicate that except Hg, spatial variability (*C*_0_) values brought by measuring error, microscale process and other random parts were all greater than structure variance, i.e., *C*_0_/(*C*_0_ + *C*_1_) values were all greater than 50%, and random factors played a dominant role. Spatial variability of heavy metals mainly stems from soil covering, improvement of soil fertility, soil pH conditioning measures and random factors.

### Fractal Analysis

**Figure 3:**
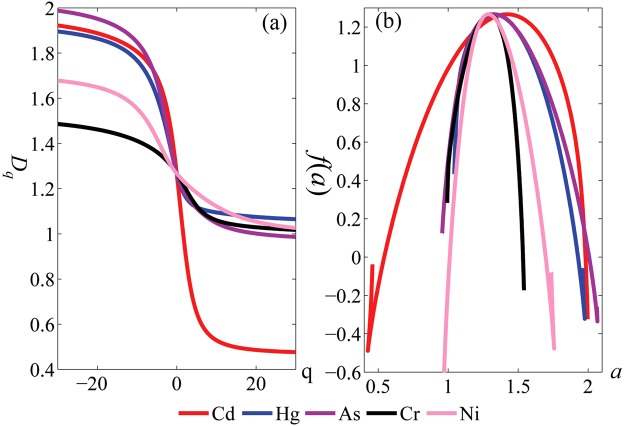
Rayleigh spectrum (**a**) and multifractal spectrum (**b**) of measured values for different heavy metal. Note: *q* is given real number, *f*(*α*) is the multifractal spectrum, and *α* singular exponent.

The multifractal analysis was used to calculate fractal parameters (Table 2), and to draw corresponding Rayleigh spectrum and multifractal spectrum of different heavy metals in reclaimed soil (Fig. 3). The above figures and related parameters may quantitatively and intuitively reveal the local characteristics of multifractal such as spatial heterogeneity, heterogeneity and shape characteristics of heavy metals in reclaimed soil, providing a basis for determining whether spatial prediction may be conducted by Mkriging or not.

**Table 2:** Multifractal characteristics of different kinds of heavy metal.

Elements	*α*_min_	*α*_max_	*D*_*q*min_	*D*_*q*max_	Δ*α*	Δ*D*_*q*_
Cd	0.4261	1.9971	0.4762	1.9223	1.5710	1.4461
Hg	1.0438	1.976	1.0649	1.8958	0.9322	0.8309
As	0.9576	2.0663	0.9863	1.9879	1.1087	1.0016
Cr	0.9931	1.5416	1.0176	1.4863	0.5485	0.4687
Ni	0.9717	1.7574	1.0257	1.6783	0.7857	0.6526

Note: *α*_max_ and *α*_min_ is the maximum and minimum value of the singular exponent, respectively. *D*_*q*min_ and *D*_*q*max_ is the maximum and minimum value of the Rayleigh dimension, respectively, Δ*D*_*q*_ is their difference, representing the degree of variation of Rayleigh dimension. Δ*α* expresses multifractal spectrum width.

The process dividing fractal into hierarchical zones by setting *q* at different values is called multifractal analysis. In theory, the larger the value range of *q* was, the better the result of multifractal analysis will be. In actual computation, however, with the increase of value *q*, workload will be multiplied and when *q* is raised to a specific degree, *D*_*q*_, *α* and *f*(*α*) will not change with the increase of *q*. Through multiple experimental simulation and in view of related literature^28^, *q* was set at −30 ≤ *q* ≤ 30.

The larger the variation of Rayleigh dimension (Δ*D*_*q*_) is, the larger the multifractal spectrum width (Δ*α*) will be. If Rayleigh spectrum is a straight line (Δ*D*_*q*_ → 0), multifractal spectrum will be concentrated to one point and the study object will show single fractal^52^. Figure 3(a) indicated when *q* → +∞, the maximum probability plays a decisive role; when *q* → −∞, the minimum probability plays a decisive role. None of the five kinds of heavy metals in reclaimed soil showed a linear distribution, suggesting these elements have a multifractal feature and are suitable to adopt Mkriging method for spatial digital mapping^28,52^.

Figure 3(b) and Table 2 indicated Δ*D*_*q*_ of the five kinds of soil heavy metals was consistent with Δα in the multifractal spectrum. Cd has the largest Δ*D*_*q*_ (Δ*D*_*q*_ = 1.4461), followed by As (Δ*D*_*q*_ = 1.0016), Hg (Δ*D*_*q*_ = 0.8309), Ni (Δ*D*_*q*_ = 0.6526) and Cr (Δ*D*_*q*_ = 0.4687); Δα width also shows the same behavior, Cd (Δ*α*=1.571) > As (Δ*α* = 1.1087) > Hg (Δ*α* = 0.928) > Ni (Δ*α* = 0.7857) > Cr (Δ*α* = 0.5485). The wider multifractal spectrum Δ*α* means more irregular spatial distribution of heavy metals in this soil and more fractal. The five heavy metals in reclaimed soil of industrial and mining abandoned land are in the following descending order in terms of homogeneity: Cd, As, Hg, Ni and Cr.

### Digital mapping

**Figure 4:**
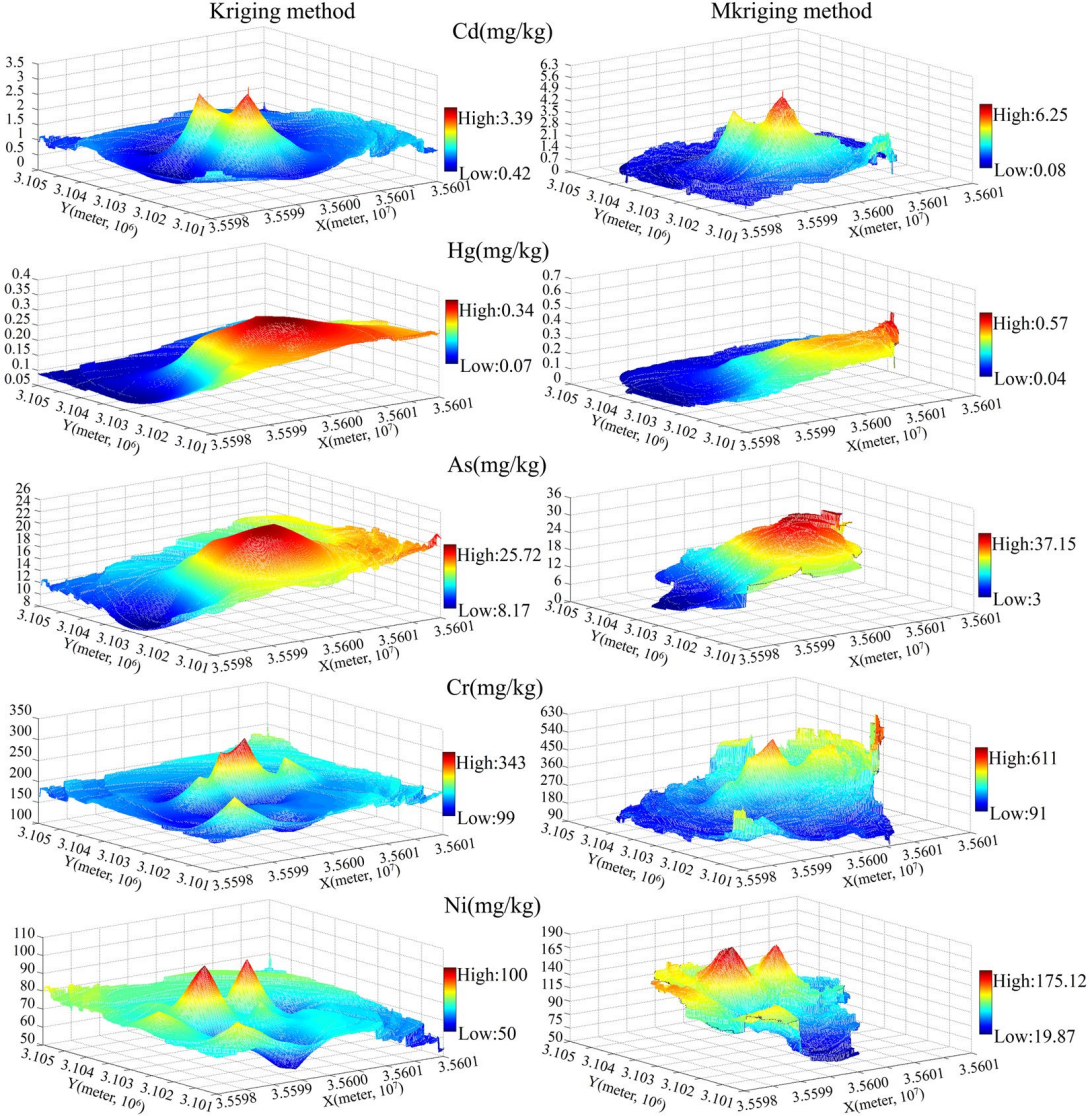
Predicted distribution of heavy metals in reclaimed soil based on kriging and Mkriging method.

Spatial mapping of heavy metal content in reclaimed soil was conducted based on ordinary Kriging and Mkriging Considering the characteristics of variation function, the setting of the search radius of Mkriging method varied accordingly. For different heavy metals in reclaimed soil, five boundary points are selected in 500~3000 m, the side length is set as 4000 m and the observation scale is 0.3~0.6 (empirical value). The output grid is 10 m × 10 m (Fig. 4).

Figure 4 revealed that regardless of spatial mapping methods, the same heavy metal in reclaimed soil basically had a consistent predicted spatial distribution pattern. The heavy metal content in reclaimed soil of the study area was high in the middle part and low in the periphery. This pattern was caused by mining, reclamation and other human activities, as well as topography, landforms and other natural factors. Damage by mining and reclamation activities were the major influence factors. Higher concentrations of heavy metals are found in areas with lower topography and more sulfur slag heap. Historically, as a subsidiary area of mining, heavy metal content was relatively low due to its high topography and far away from the sulphur slag heap.

Figure 4 indicated that based on Mkriging method, the fluctuation was stronger, and the depiction of local specific values was more meticulous. The ranges of predicted values with Mkriging of elements Cd, Hg, As, Cr and Ni in reclaimed soil were 0.08–6.25 mg/kg, 0.04–0.57 mg/kg, 3–37.15 mg/kg, 91–611 mg/kg and 19.87–175.12 mg/kg respectively, the corresponding amplitudes were 6.20 mg/kg, 0.53 mg/kg, 34.15 mg/kg, 520 mg/kg and 155.34 mg/kg respectively, close to the ranges of real samples. It was a breakthrough to the past conclusion that the prediction results by conventional methods were all a subset of measured values. Mkriging method was more capable to reflect the spatial structure of original regionalized variables and the mutual relations of variables. In comparison, the ranges of predicted values with ordinary Kriging of Cd, Hg, As, Cr and Ni were 0.42–3.39 mg/kg, 0.07–0.34 mg/kg, 8.17–25.72 mg/kg, 99–343 mg/kg and 50–100 mg/kg respectively and all were a subset of corresponding measured values. Their corresponding amplitudes were 2.97 mg/kg, 0.27 mg/kg, 17.55 mg/kg, 244 mg/kg and 50 mg/kg respectively, only 2.07, 1.96, 1.94, 2.12, and 3.11 times of corresponding elements with Mkriging method, and close to the averages of the real samples. Kriging method had obvious smoothing and central effects.

### Effect evaluation

The effects of different methods on spatial prediction of heavy metal content in reclaimed soil of industrial and mining abandoned land were comprehensively evaluated form three aspects including specific value reproduction effect, prediction accuracy and model simulation effect evaluation.

#### Comparison of reproduction effect of specific values

The difference in damage background and reclamation measures resulted in mutability and variability of spatial distribution of heavy metals. At present, the mostly frequently used spatial mapping method was ordinary Kriging method, but ordinary Kriging method alone had a smoothing effect and was unable to highlight the mutability of quality elements of reclaimed land resulting from the difference of reclamation measures. Therefore, it was crucial for a spatial digital mapping method to accurately depict the mutability of heavy metals. We used the coverage diagram and coverage ratio of specific value (CRSV) to describe the coincidence of different methods between predicted and measured specific values. We chose 20% of the minimum or maximum measured and predicted values as statistical magnitudes. CRSV value showed the proportion of their overlap.

**Figure 5:**
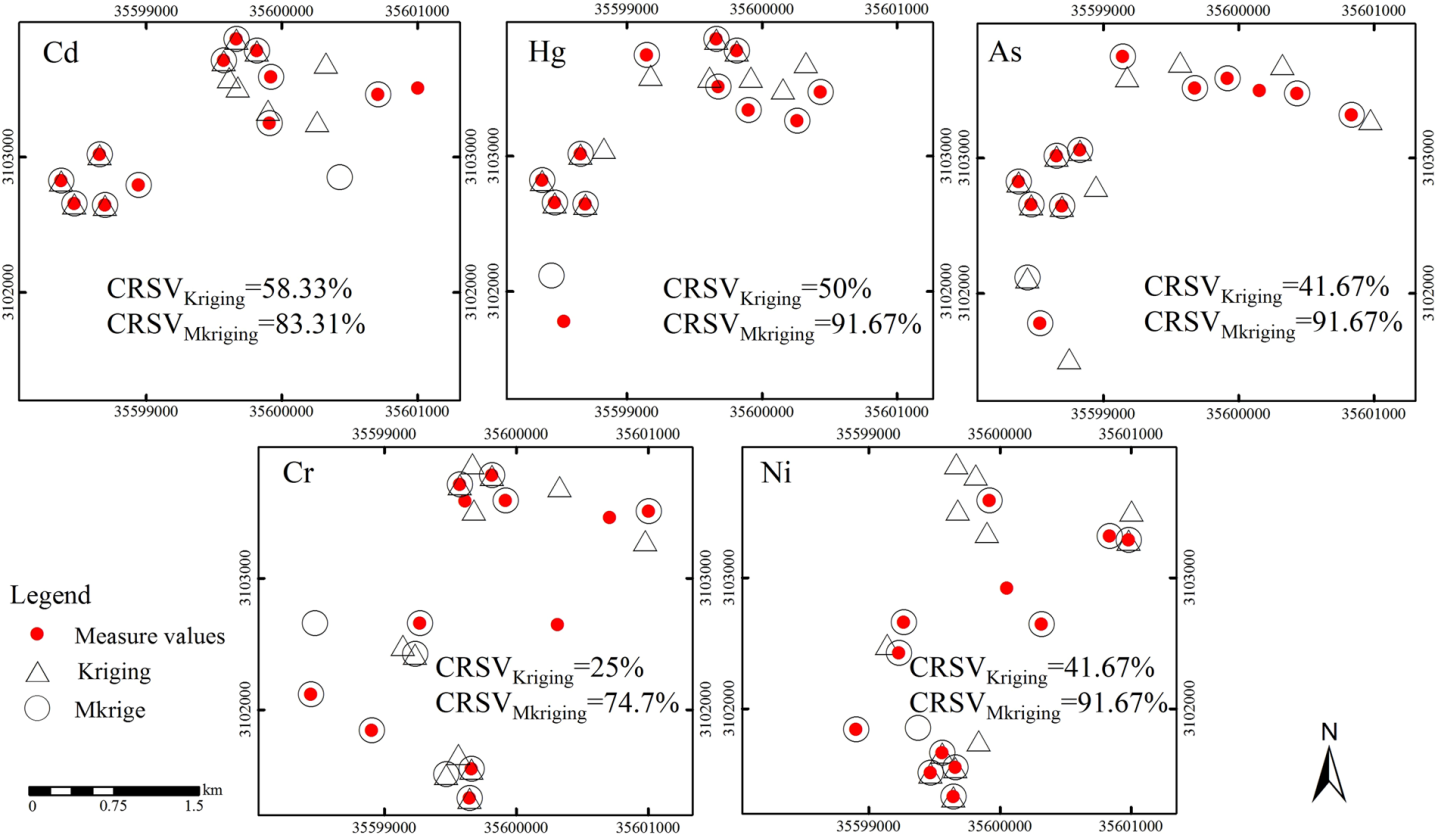
Comparison of the minimum specific values of different soil heavy metals between measured and predicted values under different methods. Note: Red dots stand for specific values of 20% minimum measured values, black circles stand for the minimum values of 20% of predicted values based on Mkriging, and black triangles stand for the minimum values of 20% of the predicted values based on kriging. CRSV value showed the proportion of their overlap.

The minimum CRSV value of the same heavy metal element in reclaimed soil showed obvious differences among different methods. Regardless of the types of heavy metals in reclaimed soil, the minimum CRSV values with Mkriging method were 83.31%, 91.67%, 91.67%, 74.7% and 91.67% for five heavy metals Cd, Hg, As, Cr and Ni, respectively (Fig. 5). The measured and predicted values of Hg, As and Ni had the highest degree of coincidence. The predicted values based on Mkriging could more effectively reproduce the minimum specific values of the measured values. In comparison, kriging method showed a lower degree of coincidence in the minimum specific values of different heavy metals, and the CRSV value of element Cd did not exceed 60% though highest, while the lowest CRSV of element Cr was only 25%.

Heavy metal elements Cd, Hg and As in reclaimed soil have similar spatial distribution patterns, and showed a feature of being concentrated and mainly distributed in the western part and northern part of the area, i.e., in the perimeter zone of the study area, consistent with the pattern of the spatial distribution diagram as shown in Fig. 4. Before reclamation, these regions were mostly the land for auxiliary facilities such as factories and dormitories of mining and the pollution was less serious. During reclamation, soil covering and other protective measures were adopted.

**Figure 6:**
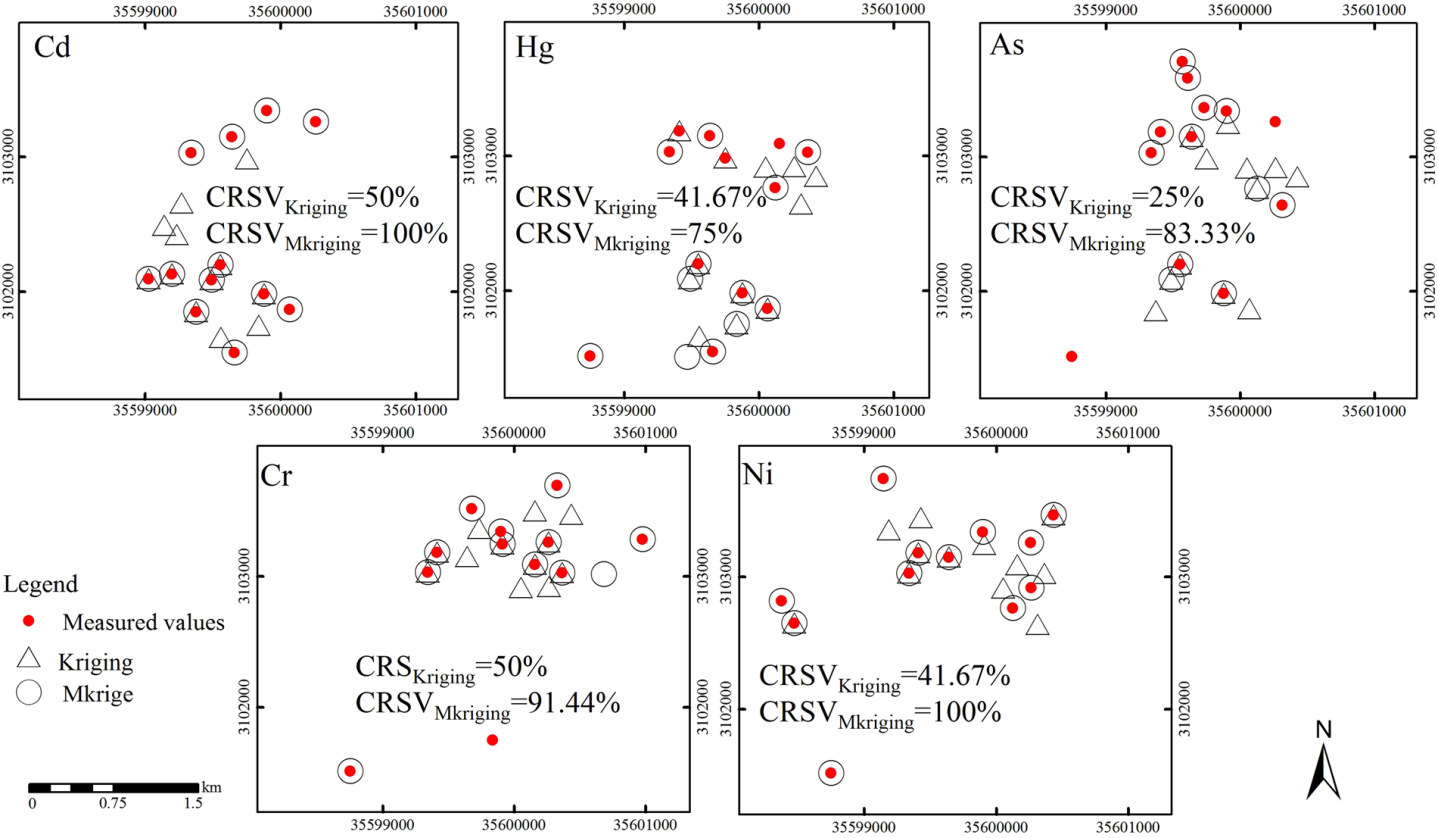
Comparison of the maximum specific values of different soil heavy metals between measured and predicted values under different methods. Note: Red dots stand for specific values of 20% maximum measured values, black circles stand for the maximum values of 20% of predicted values based on Mkriging, and black triangles stand for the maximum values of 20% of the predicted values based on kriging. CRSV value showed the proportion of their overlap.

There are greater differences between the two spatial prediction methods on the reproduction of the maximum specific value (Fig. 6). The maximum predicted values of elements Cd and Ni fully coincide with measured values, and the CRSV value was as high as 100%. The CRSV values were 100%, 75%, 83.33%, 91.44% and 100% respectively, while those of heavy metal elements Cd, Hg, As, Cr and Ni in soil based on ordinary Kriging were 50%, 41.67%, 25%, 50% and 41.67% respectively, mostly only one half of the values based on Mkriging method. The CRSV value of element As was less than one third of the value based on Mkriging method. As far as the reproduction of prediction results to specific values (mutability) was concerned, the spatial digital mapping of heavy metals in reclaimed soil of industrial and mining abandoned land based on Mkriging was more scientific and comprehensive.

#### Comparison of prediction accuracy and model simulation effect

Full-sample cross RMSE and MSDR of different heavy metals under different methods were calculated. Ordinary Kriging was chosen as a contrast method. The relative improvement (RI) values of different heavy metals based on Mkriging were calculated by Eq. 15.

15 $${\rm{RI}}=\frac{{{\rm{RMSE}}}_{{\rm{Mkrige}}}-{{\rm{RMSE}}}_{{\rm{OK}}}}{{{\rm{RMSE}}}_{{\rm{OK}}}}$$

where RMSE_Mkriging_ is RMSE of Mkriging method, and RMSE_OK_ is RMSE of the control method.

**Figure 7:**
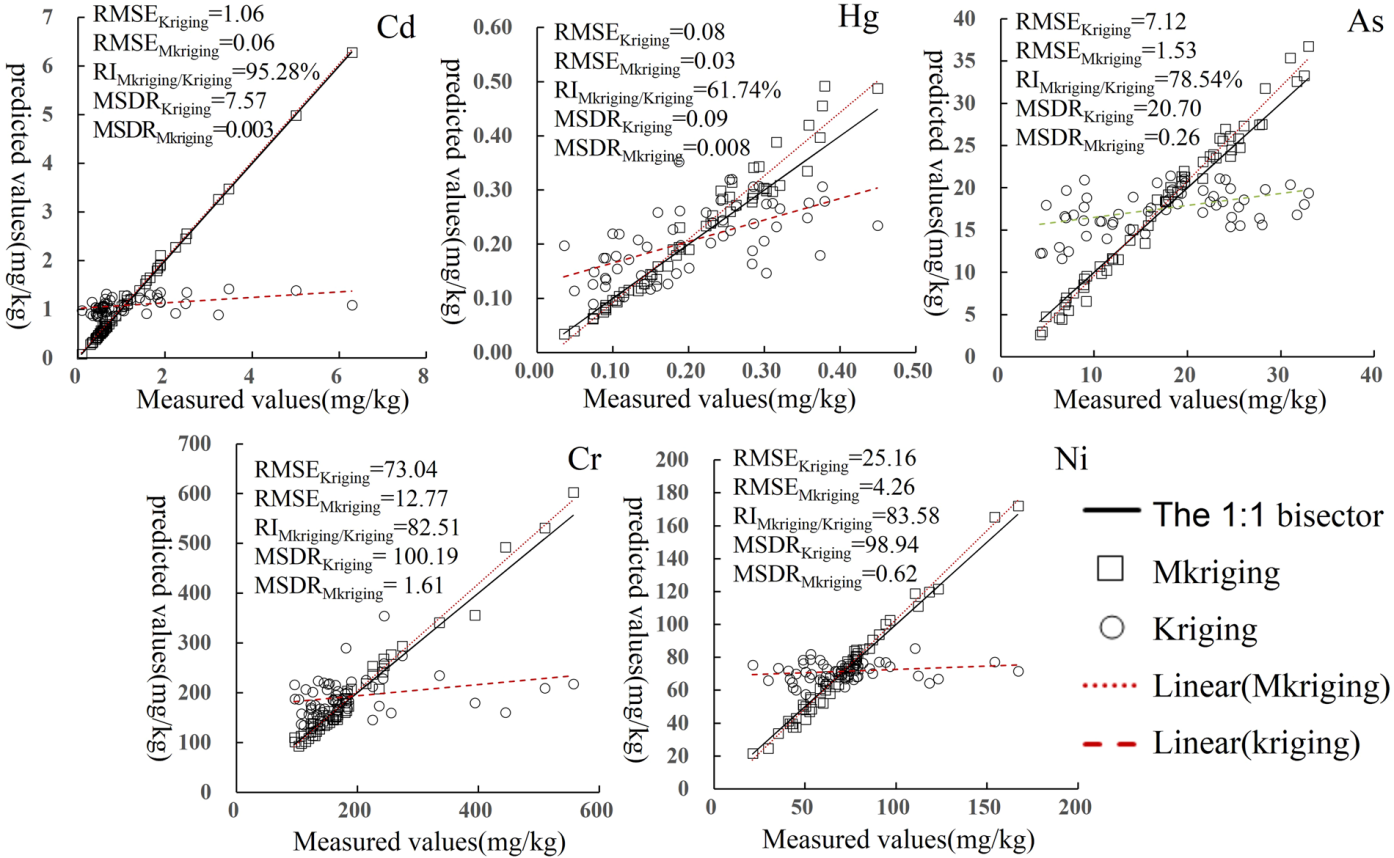
Comparison of prediction accuracy and model fitting of different soil heavy metal under different methods. Note: RMSE and MSDR was root mean squared errors and mean squared deviation ratio.

Scatter diagrams of the measured and predicted values of different heavy metals in reclaimed soil under different methods were drawn (Fig. 7) and the linear relations of predicted values under different methods were fit to compare the degree of deviation from 1:1 bisector^43,45,46^.

Figure 7 indicated that the same method showed different prediction accuracy and model fitting effects among different heavy metals in reclaimed soil, and the two methods showed a more significant difference in spatial prediction accuracy and model fitting effect of a same heavy metal in reclaimed soil. Regardless of the types of heavy metals, the predicted values based on kriging method all tended to be the same (close to the average of original data) and the fitted lines tended to be parallel, i.e., showed central and smoothing effects. In addition, the fitted lines of the predicted values of heavy metals in reclaimed soil based on kriging were blow 1:1 bisector. A larger included angle (acute angle) between the fitted line and bisector meant a greater downward deviation. When it was close to horizon, the prediction effect was the poorest. The fitted line of element Ni was basically parallel with the x-axis, and the predicted values tend to the same. Mkriging method reproduces specific values more satisfactorily^28,30^, the fitted line is closer to 1:1 bisector, and the fitted line of Cd basically coincides with the bisector. The fitted lines of predicted values of all heavy metals in reclaimed soil with Mkriging method were all above 1:1 bisector, and predicted values were generally larger, but the degree of deviation was much smaller than OK method. The relation between the fitted curve and bisector also revealed Mkriging was more capable to reflect the spatial structure of original regionalized variables and the mutual relations of variables.

The RMSE (RMSE_Cd_ = 1.06, RMSE_Cd_ = 0.08, RMSE_Cd_ = 7.12, RMSE_Cd_ = 73.04, and RMSE_Cd_ = 25.16) and MSDR (MSDR_Cd_ = 7.57, MSDR_Cd_ = 0.09, MSDR_Cd_ = 20.70, MSDR_Cd_ = 100.19, and MSDR_Cd_ = 98.94) values with Kriging were larger, which indicated that the prediction accuracy is low and the fitting effect is poor. Mkriging was just the opposite, The RMSE and MSDR values were generally smaller, showing higher prediction accuracy and a better model fitting effect (Fig. 7). Compared with Kriging method, the RIs of RMSE of elements Cd, Hg, As, Cr and Ni with Mkriging were 95.28%, 61.74%, 78.54%, 82.51% and 83.58% respectively, and the spatial prediction accuracy of heavy metal element Cd in reclaimed soil based on Mkriging method is almost doubled.

Combining CRSV, RMSE and MSDR can not only reflect the characteristics of original samples and maintain the spatial variability and mutability of original samples but also guarantee prediction accuracy and model fitting effect of different methods. After comprehensive consideration of reproduction of specific values, RMSE and MSDR as well as measured and predicted scatter diagrams, the effect of spatial prediction based on Mkriging method was obviously higher than OK method for soil heavy metals in the study area. OK method was a low-pass filter and unable to rebuild the high frequency, local and weak signals of original signals^28,50^. The deletion of local variation information may result in the loss of some useful data^28,48,50^.

Mkriging method had a better overall effect, which may highlight specific values, and more satisfactorily maintain and reproduce the spatial structure of original data-based variables^28,48^. The effect of spatial prediction with Mkriging also showed certain difference among different kinds of heavy metals, and element Cd showed the best effect, followed by Hg, As, Ni and Cr, which was consistent with the fractal degrees, that was to say, the larger the fractal degree is, the more accurate the prediction will be. Element Cr showed a relatively poor prediction effect because it probably had a feature of tending to single fractal. Mkriging method may be used as one of the scientific digital mapping methods for heavy metals in reclaimed soil of industrial and mining abandoned land.

## Conclusions and Discussions

We revealed the advantages of Mkriging method, provides methodological guidance for spatial prediction of heavy metal content in reclaimed soil of industrial and mining abandoned land.

1. For reasons of history and reclamation, the original samples of heavy metals in reclaimed soil of industrial and mining abandoned land showed a very large range and variation degree, the *C*_0_/(*C*_0_ + *C*_1_) values of different heavy metals basically were all greater than 50%, random factors played a dominant role, and spatial variability mainly steamed from soil covering, conditioning measures of soil pH and other random factors. Such characteristics of original data decided the Kriging method cannot be used for spatial digital mapping.
2. Based on Mkriging method, the fluctuation was stronger, the depiction of local specific values was more meticulous and closer to the ranges of real samples of heavy metals in reclaimed soil. The Mkriging method was more capable to reflect the spatial structure of original regionalized variables and the mutual relations of variables. In comparison, the predicted values with Kriging are closer to the average of all real samples of heavy metals in reclaimed soil and had obvious smoothing and central effects.
3. After comprehensive consideration of CRSV, RMSE and MSDR as well as measured and predicted scatter diagrams, regardless of the types of heavy metals in soil, the effect of spatial digital mapping with Mkriging was obviously higher than Kriging method. Mkriging method may be used as one of the scientific methods predicting elements Cd, Hg, As, Cr and Ni and other heavy metals in reclaimed soil of industrial and mining abandoned land. Mkriging method may highlight specific values, and more satisfactorily maintain and reproduce the spatial structure of original data-based variables. The larger the fractal degree of a heavy metal in reclaimed soil is, the more accurate the prediction will be.
  The Mkriging method may interpolate irregularly distributed time-space signals into regularly distributed signals, which may extract the high frequency, local and weak signals of spatial signals. It was estimated that the process parameters may be used as characteristic parameters for mode recognition. The convolution filtering theory was applied to quantitatively derive the low-pass filtering feature of geostatistics. During interpolation, high frequency, local and weak signals were lost. After defining the measurement scale and measure of spatial signals, the article realized multidifractal interpolation, which retained more high frequency information in the system^56,57^.
4. The combination of CRSV with RMSE and MSDR is an effective way to evaluate the effect of spatial mapping, which can not only reflect the spatial variability and mutability of original samples but also clearly state the prediction accuracy and model fitting effect of different methods.
5. The analysis of this article also indicated that Mkriging showed a good overall prediction effect of heavy metals in reclaimed soil of industrial and mining abandoned land, but its prediction effect on element Cr was poor probably due to the small sample size. A more suitable method needed to be selected in combination with other relevant methods, including Empirical Bayes Kriging method.
